# Alendronate Can Improve Bone Alterations in Experimental Diabetes by Preventing Antiosteogenic, Antichondrogenic, and Proadipocytic Effects of AGEs on Bone Marrow Progenitor Cells

**DOI:** 10.1155/2016/5891925

**Published:** 2016-10-20

**Authors:** Sara Rocío Chuguransky, Ana María Cortizo, Antonio Desmond McCarthy

**Affiliations:** Laboratorio de Investigaciones en Osteopatías y Metabolismo Mineral (LIOMM), Department of Biological Sciences, School of Exact Sciences, National University of La Plata, Calle 47 y 115, 1900 La Plata, Argentina

## Abstract

Bisphosphonates such as alendronate are antiosteoporotic drugs that inhibit the activity of bone-resorbing osteoclasts and secondarily promote osteoblastic function. Diabetes increases bone-matrix-associated advanced glycation end products (AGEs) that impair bone marrow progenitor cell (BMPC) osteogenic potential and decrease bone quality. Here we investigated the* in vitro* effect of alendronate and/or AGEs on the osteoblastogenic, adipogenic, and chondrogenic potential of BMPC isolated from nondiabetic untreated rats. We also evaluated the* in vivo *effect of alendronate (administered orally to rats with insulin-deficient Diabetes) on long-bone microarchitecture and BMPC multilineage potential.* In vitro*, the osteogenesis (Runx2, alkaline phosphatase, type 1 collagen, and mineralization) and chondrogenesis (glycosaminoglycan production) of BMPC were both decreased by AGEs, while coincubation with alendronate prevented these effects. The adipogenesis of BMPC (PPAR*γ*, intracellular triglycerides, and lipase) was increased by AGEs, and this was prevented by coincubation with alendronate.* In vivo*, experimental Diabetes (a) decreased femoral trabecular bone area, osteocyte density, and osteoclastic TRAP activity; (b) increased bone marrow adiposity; and (c) deregulated BMPC phenotypic potential (increasing adipogenesis and decreasing osteogenesis and chondrogenesis). Orally administered alendronate prevented all these Diabetes-induced effects on bone. Thus, alendronate could improve bone alterations in diabetic rats by preventing the antiosteogenic, antichondrogenic, and proadipocytic effects of AGEs on BMPC.

## 1. Introduction

Bisphosphonates (BPs) have been extensively used to treat postmenopausal, age-related and steroid-induced osteoporosis, Paget's disease, and osteolysis-induced hypercalcemia associated with multiple myeloma and metastatic cancers [1]. The chemical structure of these drugs is similar to inorganic pyrophosphate: while the latter has a P–O–P bond linking both phosphate groups, in the former it is replaced by a P–C–P structure. BPs possess a so-called “bone hook” consisting of both phosphonate groups, facilitating its binding to bone mineral [2]. Thus, BPs accumulate rapidly in bone tissue where they primarily inhibit the activity of bone-resorbing osteoclasts [1].

Nitrogen-containing bisphosphonates (N-BPs) such as alendronate are the most potent kind; they inhibit farnesyl pyrophosphate synthase (FPPS), a key regulatory enzyme in the mevalonate pathway, and thereby prevent prenylation of small GTPase signaling proteins. In bone, protein isoprenylation is essential for osteoclastogenesis and bone resorption [1, 3]. Thus, treatment with N-BPs is currently the most common treatment for postmenopausal osteoporosis [1, 3].* In vivo*, treatment with BPs has an antifracture effect via inhibition of bone resorption and increase in bone mass [3].

Previous studies suggest that nonresorbing bone cells such as osteoblasts and osteocytes may also be targets of BPs action [4]. It has been reported that BPs can inhibit glucocorticoid-induced apoptosis in these bone cells, a process that appears to be mediated by Cx43 hemichannel aperture [5]. In fact, Cx43 is required for the antiapoptotic effect of BPs on bone cells, both* in vitro* and* in vivo* [6, 7].

During aging and postmenopausal osteoporosis there is an increase in osteoclastic activity, as well as a shift in the progression of bone marrow progenitor cells (BMPC) towards an adipocytic phenotype in detriment of osteoblastogenesis [8]. This selection of adipogenesis over osteoblastogenesis in the bone marrow microenvironment has also been reported in other conditions such as type 1 Diabetes mellitus, in which there is an accumulation of bone matrix advanced glycation end products (AGEs) [9, 10]. A crucial step in the formation of mineralized tissue involves osteoblastic commitment of BMPC, followed by their mobilization to the bone surface. Maintenance of bone cell homeostatic balance depends on adequate cell-matrix and cell-cell interactions [11]. We have previously shown that high levels of AGEs accumulated on bone collagen can decrease the integrin-mediated attachment of osteoblasts to the matrix [12]. We have also found that soluble and matrix-associated AGEs can impair osteoblastic growth and differentiation via activation of RAGE (receptor for AGEs), potentially contributing to diabetic osteopenia [13, 14]. Thus, accumulation of bone AGEs has been implicated in a Diabetes-induced decrease in bone quality and/or mass, leading to an increase in fracture risk [15, 16].

An increase in the glycation of bone matrix proteins could be associated not only with loss of bone quality, but also with differential responses to antiosteoporotic treatments. The use of BPs for treatment of diabetic osteopathy is still an unsolved issue. Although some authors have reported a BPs-induced improvement in bone mineral density (BMD) of diabetic postmenopausal women [17, 18], other researchers have suggested that postmenopausal women with type 2 Diabetes could be resistant to a long-term treatment with BPs [19]. Interestingly, we have previously found that low doses (10^−8^ M) of alendronate can completely prevent the* in vitro* antiosteogenic and proapoptotic effects of AGEs on osteoblastic cells [20]. This effect of alendronate is blocked by nifedipine, an L-type calcium channel inhibitor. Although this data provides evidence suggesting that BPs could have indirect anabolic actions on osteoblasts (i.e., blocking AGEs-induced deleterious effects on this cell type), the effect of alendronate on BMPC multilineage progression in the context of an increase in bone AGEs (such as diabetic bone) remains unknown.

In the present study we have evaluated the* in vitro* effect of alendronate and/or AGEs on the osteoblastogenic, adipogenic, and chondrogenic potential of BMPC obtained from control rats. We have also investigated the* in vivo* effect of alendronate on long-bone microarchitecture in rats with insulin-deficient Diabetes, as well as the* ex vivo* action of this bisphosphonate on BMPC multilineage progression (i.e., the effect of orally administered alendronate on the osteogenic, adipocytic, and chondrogenic potential of BMPC obtained* ex vivo* from treated animals). In addition, possible mechanisms of action have been studied.

## 2. Materials and Methods

### 2.1. Materials

Alendronate [1-hydroxy-3-aminobutylidene-1, 1-bisphosphonic acid] was kindly provided by Dr. Hector Ostrowski of Elea Laboratories (Argentina). Dulbecco's modified Eagle's medium (DMEM) was obtained from Invitrogen (Buenos Aires, Argentina), trypsin–EDTA was from Gibco and fetal bovine serum was obtained from Natocor (Córdoba, Argentina). Tissue culture disposable material was from Nunc (Tecnolab, Buenos Aires, Argentina). Centricon 10 kDa cutoff filter cartridges were purchased from Amicon Inc. (Beverly, MA, USA). Bovine serum albumin, D-glycolaldehyde, Triton X-100 and Sirius Red dye were obtained from Sigma-Aldrich (Buenos Aires, Argentina). Dihydrorhodamine 123 (DHR) was from Molecular Probes (Buenos Aires, Argentina). All other chemicals and reagents were purchased from commercial sources and were of analytical grade.

### 2.2. Preparation of Advanced Glycation End Products

AGEs-modified bovine serum albumin (BSA) was prepared by incubation of 10 mg/mL BSA with 33 mM D-glycolaldehyde in 150 mM phosphate-buffered saline pH 7.4 at 37°C for 3 days under sterile conditions, as we have previously described [20]. Control BSA was incubated in the same conditions without sugar. Unincorporated sugar was removed by centrifugation/filtration with Centricon filter cartridges. The formation of AGEs was assessed by fluorescence emission at 420 nm upon excitation at 340 nm. The estimated levels of AGEs-BSA obtained by this* in vitro* incubation were 16.9% relative fluorescence intensity/mg protein, in contrast to 3.7% for control BSA.

### 2.3. Animal Treatments

Three-month-old male Sprague-Dawley rats (190–210 g) were used. Animals were maintained in a temperature-controlled room at 23°C, with a fixed 12 h light: 12 h darkness cycle, and fed standard rat laboratory chow and water ad libitum. All experiments on animals were done in conformity with the Guidelines on Handling and Training of Laboratory Animals published by the Universities Federation for Animals Welfare [21]. Approval for animal studies was obtained from our institutional animal welfare committee (Protocol Number 001-05-15).

For* in vitro* studies, control (nondiabetic untreated) animals were used as a source of BMPC. Briefly, they were sacrificed under anaesthesia by rapid cervical dislocation. Bone marrow cells were collected by flushing the femoral diaphysis medullary canal with Dulbecco's modified essential medium (DMEM) under sterile conditions and processed as described in the following sections.

To perform* in vivo* and* ex vivo* studies, 16 animals were submitted to i.p injection of nicotinamide (50 mg/kg in physiological saline) followed by i.p. streptozotocin (60 mg/kg freshly dissolved in 0.05 M citrate buffer pH 4.5) in order to induce partially insulin-deficient Diabetes mellitus [22, 23], while another group of 16 rats was allowed to remain nondiabetic. A week later, nonfasting blood glucose (Wiener, Rosario, Argentina) and insulin (ALPCO, USA) were assayed according to instructions of the manufacturer, in order to verify the metabolic status of animals. Control animals showed the following results: 1.35 ± 0.15 ng/mL for insulin and 171 ± 8 mg/dL for glucose. Results for diabetic animals were 0.25 ± 0.09 ng/mL for insulin and 433 ± 22 mg/dL for glucose. Rats were then subdivided into four groups of eight animals per group: control (nontreated nondiabetic) (C) and untreated diabetic rats (D) received water* ad libitum*, whereas alendronate-treated nondiabetic (CA) and alendronate-treated diabetic (DA) rats received 1 mg/kg/day of alendronate (Elea Lab., Buenos Aires, Argentina) in their drinking water for 2 weeks. After all treatments, nonfasting blood samples were taken and serum was stored at −20°C until biochemical evaluation. Commercial kits were used to measure serum glucose, triglycerides, cholesterol, and fructosamine. All rats were sacrificed under anaesthesia by rapid cervical dislocation, after which one femur of each animal was processed for BMPC isolation and the other for histomorphometric evaluation, as described in the following sections.

### 2.4. Histomorphometric Examination of Long Bones

In order to evaluate the possible* in vivo* effect of alendronate on long bones from diabetic and nondiabetic animals, femora were processed for quantitative histomorphometric analysis. Dissected bones were fixed in 10% formalin and decalcified in 10% EDTA, embedded in paraffin, and 5 *μ*m sections were obtained with an SM 2000R Leica microtome. The sections were stained with either tartrate-resistant acid phosphatase histochemistry (TRAP) (Sigma, Buenos Aires, Argentina) to specifically identify osteoclasts or hematoxylin-eosin (H-E). Pictures were taken with a Nikon Coolpix 4500 digital camera on an Eclipse E400 Nikon microscope. Images were analyzed using the Image J program with a microscope scale plugin. In all experimental groups, microarchitecture of the proximal femoral metaphysis was evaluated by H-E (relative trabecular volume, bone osteocytic density, and bone marrow adipocytic density) 250 *μ*m distal from the cartilage growth plate. Osteoclastic density was determined in the secondary spongiosa and calculated as positive TRAP area per square millimeter (Oc/mm^2^).

### 2.5. BMPC Isolation and Processing

Bone marrow progenitor cells were obtained as we have described previously [24]. As stated above, BMPC for* in vitro* experiments were derived from untreated nondiabetic animals, while those for* ex vivo* studies were obtained from C, D, CA, and DA groups. In each case, unfractioned bone marrow cells were flushed out of the femoral diaphysis medullary canal with Dulbecco's modified essential medium (DMEM) under sterile conditions. The resulting suspension was seeded in a 25 cm^2^ tissue culture flask and incubated in DMEM supplemented with penicillin (100 UI/mL), streptomycin (100 mg/mL), and 10% fetal bovine serum (FBS) (Natocor, Córdoba, Argentina) (basal medium) at 37°C in a humidified atmosphere with 5% CO_2_ and 95% air. Nonadherent cells were removed by changing the medium after 24 hours. The culture medium was changed twice a week. When cells reached confluence (after 10 to 15 days), the cell monolayer was detached using 0.12% trypsin and 1 mM EDTA and subcultured in tissue culture plates. Cells were then cultured as described below in different media, in order to induce their commitment and progression in a lineage-specific manner.

In the case of* in vitro* experiments, either control BSA or AGEs-BSA (with or without 10^−8^ M alendronate) was added to each differentiation medium during the last 7 days of culture (for osteogenic and chondrogenic differentiation) or during the last 3 days (for adipogenic differentiation).

### 2.6. Osteogenic Differentiation of BMPC

BMPC were plated at a density of 5 × 10^4^ cells/well in basal medium in 24-well plates and cultured until confluence. Subsequently, they were induced to differentiate to osteoblasts using an osteogenic medium (DMEM–10% FBS containing 25 mg/mL ascorbic acid and 5 mM sodium beta–glycerol-phosphate) [24]. Osteoblastic differentiation was evaluated after 15 days of culture by measuring specific alkaline phosphatase activity (ALP) and type 1 collagen production and after 21 days by examination of extracellular calcium deposition.

For ALP evaluation [13], cell monolayers were washed with phosphate-buffered saline (PBS) and lysed with 250 *μ*L 0.1% Triton-X100. ALP was measured with a 100 *μ*L aliquot of the lysate, by its capacity to hydrolyze p-nitrophenylphosphate (p-NPP) into p-nitrophenol (p-NP) at 37°C for 1 hour. The absorbance of p-NP was recorded at 405 nm. Aliquots of the same extract were used for protein determination by Bradford's technique [25]. Type 1 collagen production was evaluated as reported previously [24]. Briefly, cell monolayers were fixed with Bouin's solution and stained with Sirius red dye for 1 hour. The stained material was dissolved in 1 mL 0.1 N sodium hydroxide, and the absorbance of the solution was recorded at 550 nm. Extracellular calcium deposits (mineralization nodules) were evaluated using Alizarin S red staining [24]. Stained calcium deposits were extracted with 1 mL 0.1 N sodium hydroxide, recording the optical density at 548 nm.

### 2.7. Adipogenic Differentiation of BMPC

BMPC were grown to 50% confluence in 24-well plates in basal medium. Differentiation to adipocytes was then induced as described previously [26]. Briefly, cells were incubated for 10 days with DMEM–10% FBS supplemented with 0.5 mM 3-isobutyl-1-methylxanthine (IBMX), 1 mM dexamethasone (Decadron, Sidus, Argentina), and 200 nM insulin (Lilly, Buenos Aires, Argentina). At the end of this culture period the cell monolayers were lysed with 0.1% Triton-X100. Intracellular triacylglyceride levels and lipase activity were measured in the lysate, using commercial kits and according to instructions of the manufacturer (Wiener, Rosario, Argentina). Aliquots of the same extract were used for protein determination by Bradford's technique [25]. At the end of the culture period in representative experiments, differentiated cells were observed with a contrast-phase microscope and photographed.

### 2.8. Chondrogenic Differentiation of BMPC

BMPC were resuspended at 10^7^ cells/mL in serum-free DMEM. A 10 *μ*L drop of this suspension was transferred to each well of a 24-well plate and was incubated for 2 hours at 37°C. After this, basal medium was added to each well and adhering cells were cultured for 24 hours. Basal medium was then discarded and chondrogenic medium was added (serum-free DMEM supplemented with 10 ng/mL TGF-b3 [Peprotech, USA], 10^−8^ M dexamethasone, insulintransferrin-selenium (ITS) supplement [Invitrogen, USA]). Cells were cultured for an additional 21 days, changing the chondrogenic medium every three days. At the end of this culture period, chondrocytic differentiation was evaluated by determination of chondroitin-sulphate glycosaminoglycan (GAGs) accumulation, as previously described [27]. Briefly, cells were fixed, stained with 0.5% Alcian blue in 0.1 N HCl, and rinsed twice with 0.1 N HCl. Cells were finally rinsed with distilled water and Alcian blue associated with matrix GAGs was extracted with 4 M guanidinium-HCl, measuring absorbance at 600 nm. In representative experiments and after staining with Alcian blue, differentiated cells were observed with a contrast-phase microscope and photographed.

### 2.9. Determination of Intracellular Reactive Oxygen Species (ROS)

As a possible mechanism of action mediating the* in vitro* effect of alendronate and/or AGEs on BMPC obtained from untreated, nondiabetic animals, we measured the intracellular generation of ROS in BMPC by oxidation of dihydrorhodamine123 (DHR) to Rhodamine123 (Rho), as we have previously described [14]. Briefly, cells were cultured for 48 hours in DMEM under different conditions. After these incubations, culture media was replaced by phenol red-free DMEM with 10 *μ*M DHR and the cells were further incubated for 4 hours. After washing with PBS, the monolayer was lysated in 0.1% Triton X-100. The concentration of oxidized product present in the cell extract (Rho) was determined with a spectrofluorometer (excitation wavelength 495 nm, emission wavelength 532 nm).

### 2.10. Western Blot Analysis

BMPC from untreated, nondiabetic animals were grown to confluence in 6-well plates in DMEM–10% FBS and then induced to differentiate to either osteoblasts or adipocytes as described above (in the presence of either BSA or AGEs-BSA, with or without 10^−8^ M alendronate in the culture media). At the end of appropriate culture periods (15 days for osteogenic induction, 10 days for adipocytic differentiation), cells were lysed in Laemmli's buffer [28]. Lysates were heated to 100°C for 3 minutes, and aliquots containing 30 mg protein were subjected to 12% SDS-PAGE. Separated proteins were transferred to PVDF membranes. After washing and blocking, the membranes were incubated overnight at 4°C with an antibody specific for Cbfa1/Runx2 (Santa Cruz Biotechnology, Santa Cruz, CA, USA) for evaluation of osteoblastogenesis or against PPAR*γ* (Santa Cruz Biotechnology, Santa Cruz, CA, USA) for evaluation of adipogenesis. In order to normalize results, all blots were stripped and reprobed with an anti-beta-actin antibody (Sigma, St. Louis, MO, USA). Blots were developed by an enhanced chemiluminescence method. The intensity of the specific bands was quantified by densitometry after scanning of the photographic film. Images were analyzed using the Scion beta 2 program ImageJ program (Scion Corporation, Frederick, MD, USA).

### 2.11. Statistical Analysis

Results are expressed as the mean ± standard error of the mean (SEM) and were obtained from three separate experiments performed in sextuplicate. Differences between the groups were assessed by one-way analysis of variance (ANOVA) using the Tukey post hoc test. For nonnormally distributed data, the nonparametric Kruskal-Wallis test with the Dunn post hoc test was performed using GraphPad In Stat version 3.00 (Graph Pad Software, San Diego, CA, USA). *p* < 0.05 was considered significant for all statistical analyses.

## 3. Results

### 3.1. *In Vitro* Effects of AGEs and Alendronate on BMPC Multilineage Differentiation

As described above, in this first series of* in vitro* experiments, we evaluated the possible effects of AGEs-BSA and/or alendronate on the osteogenic, chondrogenic, and adipogenic differentiation of BMPC obtained from untreated, nondiabetic animals.

**Figure 1:**
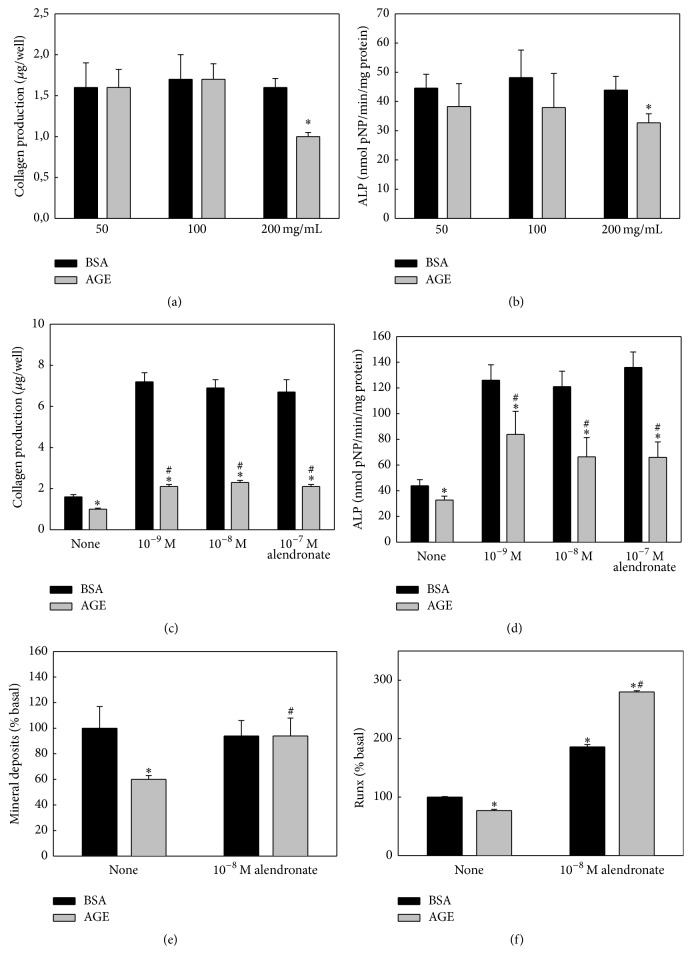
Effect of AGEs and/or alendronate on BMPC osteoblastic differentiation. BMPC were obtained from nondiabetic untreated animals and cultured in an osteogenic medium with the indicated concentrations of control BSA or AGEs-BSA (200 mg/mL if not indicated), with or without different doses of alendronate (as indicated, or 10^−8^ M if not indicated), for either 15 days (a–d and f) or 21 days (e). Type 1 collagen production (a and c), alkaline phosphatase activity (b and d), extracellular nodules of mineralization (e), and expression of Runx2 by Western blot (f) were evaluated. Results are expressed as the mean ± SEM. (a and b) ^*∗*^ *p* < 0.05 versus 50 mg/mL control BSA; (c–f) ^*∗*^ *p* < 0.05 versus control BSA, ^#^ *p* < 0.05 versus AGEs-BSA.

To begin with, we evaluated the effect of different concentrations of AGEs-BSA on the osteogenic potential of BMPC. Cells were cultured for one week in an osteogenic medium and then incubated for another week in the osteogenic medium plus different doses of either control BSA or AGEs-BSA. At the end of this incubation period, two markers of osteoblastic differentiation (type 1 collagen production and ALP activity) were evaluated. As can be seen in Figures 1(a) and 1(b), 200 mg/mL of AGEs significantly decreased both parameters. Therefore, in subsequent experiments we adopted this concentration; using a similar experimental design, BMPC were submitted to a 15- or 21-day osteogenic differentiation, in which the medium was supplemented with either 200 mg/mL control BSA or AGEs-BSA in the absence or presence of alendronate (10^−9^, 10^−8^ or 10^−7^ M) during the last week. 15-day cultures were used to evaluate type 1 collagen and ALP, and nodules of mineralization were analyzed in the 21-day cultures. Figures 1(c)–1(e) show that for the complete range of tested concentrations, both collagen production and ALP were stimulated by alendronate (Figures 1(c) and 1(d)). In addition, incubation of BMPC with AGEs-BSA inhibited their ALP and collagen production at all tested alendronate concentrations. However, although AGEs reduce collagen and ALP in the presence of alendronate, their levels in these conditions are still significantly greater than those observed for control BSA without alendronate. After 21 days of osteogenic induction AGEs-BSA significantly inhibited matrix mineralization and this effect was completely prevented by 10^−8^ M alendronate (Figure 1(e)). After 15 days of osteogenic differentiation in the conditions described above, we also evaluated the expression of the osteoblastic transcription factor Runx2 by Western blot. As shown in Figure 1(f), AGEs-BSA on its own induced a decrease in Runx2 expression and coincubation with alendronate prevented this antiosteogenic effect. AGEs-BSA appeared to enhance the effect of alendronate on Runx2 expression.

**Figure 2:**
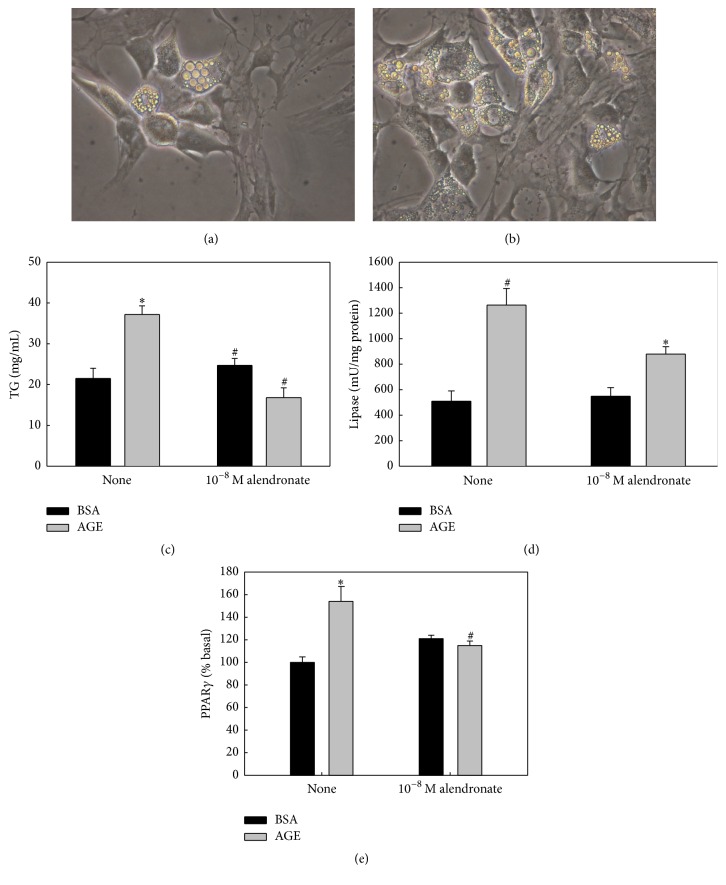
Effect of AGEs and/or alendronate on BMPC adipocytic differentiation. BMPC were obtained from nondiabetic untreated animals and cultured for 10 days in an adipogenic medium with 200 mg/mL of either control BSA or AGE-BSA, in the presence or absence of 10^−8^ M alendronate. Contrast-phase images show intracellular lipid droplets of the monolayer exposed to control BSA (a) or AGEs-BSA (b) (Obj. 40x). Intracellular triglyceride accumulation (TG) (c), lipase enzymatic activity (d), and expression of PPAR-*γ* by Western blot (e) were evaluated as markers of adipogenic differentiation. Results are expressed as the mean ± SEM. ^*∗*^ *p* < 0.05 versus control BSA; ^#^ *p* < 0.05 versus AGEs-BSA.

In another set of experiments, BMPC were induced to differentiate towards an adipocytic phenotype. The adipogenic medium was added with either control BSA or AGEs-BSA (200 mg/mL) and/or 10^−8^ M alendronate. Contrast-phase microphotographs (Figures 2(a) and 2(b)) show that exposure of BMPC to AGEs-BSA increased their accumulation of lipid droplets. Using quantitative methods, AGEs-BSA was also found to significantly increase both intracellular triglyceride content and lipase enzymatic activity (Figures 2(c) and 2(d)). In the same culture conditions, Western blot analysis showed that AGEs-BSA also increased the expression of the adipogenic factor PPAR-*γ* (Figure 2(e)). Coincubation with alendronate prevented all of these* in vitro* proadipogenic effects of AGEs on BMPC (Figures 2(c)–2(e)).

**Figure 3:**
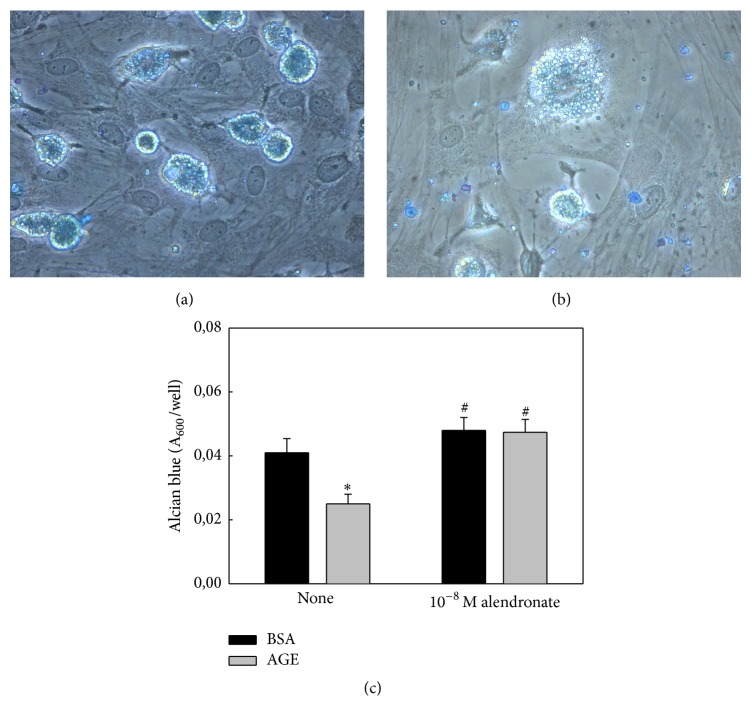
Effect of AGEs and/or alendronate on BMPC chondrocytic differentiation. BMPC were obtained from nondiabetic untreated animals and cultured for 21 days in a chondrogenic medium with 200 mg/mL of either control BSA or AGE-BSA, in the presence or absence of 10^−8^ M alendronate. Contrast-phase images of Alcian blue staining show intracellular glycosaminoglycans (GAGs) in the monolayer exposed to control BSA (a) or AGEs-BSA (b) (Obj. 40x). GAGs-associated stain was quantitated as a marker of chondrocytic differentiation (c). Results are expressed as the mean ± SEM. ^*∗*^ *p* < 0.05 versus control BSA; ^#^ *p* < 0.05 versus AGEs-BSA.

In further studies, BMPC were cultured for 21 days in a chondrogenic medium with the addition of either control BSA or AGEs-BSA, in the presence or absence of alendronate. In all cases, chondrocytic differentiation was evaluated by cell accumulation of Alcian blue-stainable glycosaminoglycans (GAG). Incubation with AGEs-BSA alone significantly decreased cell-associated GAG content. This was observed in contrast-phase microphotographs (Figures 3(a) and 3(b)) and confirmed by quantitation of GAG-associated stain (Figure 3(c)). Coincubation with alendronate prevented the* in vitro* antichondrogenic effect of AGEs on BMPC (Figure 3(c)).

### 3.2. Effect of AGEs and Alendronate on Intracellular Production of Reactive Oxygen Species (ROS)

**Figure 4:**
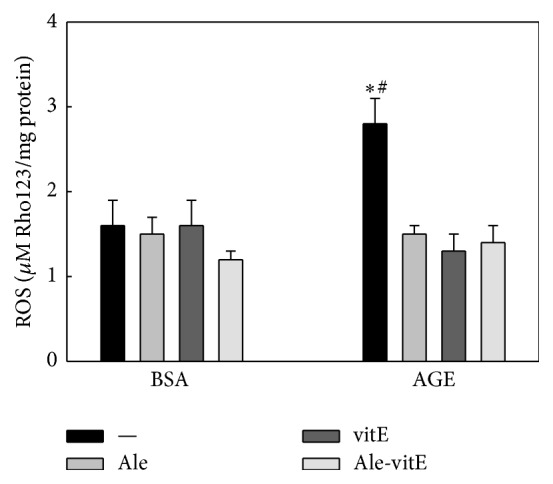
Effect of AGEs, alendronate, and vitamin E on the intracellular production of reactive oxygen species (ROS). BMPC were obtained from nondiabetic untreated animals and cultured for 48 hours with 200 mg/mL of either control BSA or AGEs-BSA, with or without 10^−8 ^M alendronate, in the presence or absence of 50 *μ*M vitamin E. ROS production was assessed by the Rhodamine123 method. Results are expressed as the mean ± SEM. ^*∗*^ *p* < 0.05 versus control BSA; ^#^ *p* < 0.05 versus BSA ± Ale, vitE, or Ale-vitE.

Intracellular generation of ROS is known to mediate certain effects of AGEs, particularly those that depend on recognition of AGEs by RAGE, the receptor for AGEs. Thus, we evaluated whether this signal transduction pathway might be involved in the* in vitro* modulation of osteoblastic differentiation by AGEs-BSA and alendronate. To this end, BMPC were incubated for 48 hours with 200 mg/mL of either BSA or AGEs-BSA with or without 10^−8^ M alendronate and in the presence or absence of 50 *μ*M vitamin E (as an antioxidant). After this incubation period, intracellular ROS production was determined by the dihydrorhodamine123 method.  Figure 4 shows that although intracellular ROS production was significantly greater in the presence of AGEs-BSA alone, this AGEs-induced increase was completely prevented by coincubation with vitamin E, alendronate, or both.

### 3.3. Diabetes-Induced Alterations in Long-Bone Microarchitecture Are Prevented by Oral Treatment with Alendronate

Insulin-deficiency was induced in rats by successive injections of streptozotocin and nicotinamide [22, 23]. Evaluation of their biochemical metabolic profile confirmed the development of Diabetes; an increase in glycaemia, fructosamine, and triglyceridemia was observed (Table 1). After induction of Diabetes the animals were treated orally with alendronate, and this did not significantly modify serum biochemical parameters (Table 1).

**Figure 5:**
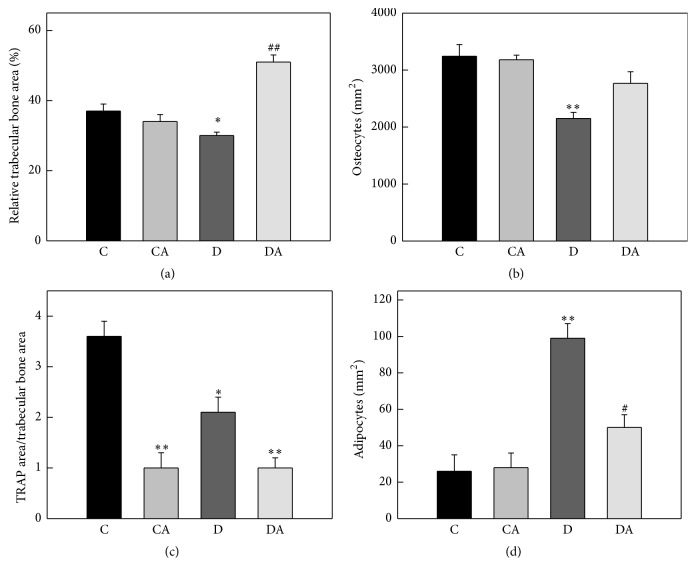
Effects of insulin-deficient Diabetes and oral treatment with alendronate on femoral microarchitecture. Diabetic and nondiabetic (control) animals were either left untreated (groups D and C) or treated for two weeks with 1 mg/kg/day of alendronate in their drinking water (groups DA and CA). Femora from animals of all groups were dissected, processed, and stained with hematoxylin-eosin or tartrate-resistant acid phosphatase histochemistry (TRAP). Proximal metaphysis was analyzed by quantitative histomorphometry to determine the following: (a) trabecular bone area, (b) trabecular osteocyte density, (c) relative osteoclastic TRAP activity in the secondary spongiosa, and (d) bone marrow adipocyte density. Results are expressed as the mean ± SEM. ^*∗*^ *p* < 0.05 versus C; ^*∗∗*^ *p* < 0.001 versus C; ^#^ *p* < 0.01 versus D; ^##^ *p* < 0.001 versus D.

The effect of Diabetes and/or alendronate treatment on femoral microarchitecture was evaluated by quantitative histomorphometric analysis. Decalcified bone sections were stained with H-E and evaluated in the secondary spongiosa to determine relative trabecular area and osteocyte density, as well as bone marrow adipocyte density. In addition, TRAP staining was performed to evaluate osteoclasts on bone surfaces of the secondary spongiosa. Compared to femora from control rats, those of diabetic rats exhibited a significant decrease in their trabecular area, osteocytic density, and TRAP activity, together with a significant increase in the density of bone marrow adipocytes (Figures 5(a)–5(d)). Alendronate treatment of diabetic animals completely prevented the Diabetes-induced decrease in trabecular bone area and osteocyte number, and it partially prevented the increase in bone marrow adiposity. As expected, alendronate treatment greatly inhibited TRAP activity (Figure 5).

### 3.4. Diabetes-Induced Alterations in BMPC Phenotypic Commitment Are Prevented by Oral Treatment with Alendronate

The effects of insulin-deficient Diabetes and/or oral treatment with alendronate, on the osteogenic, adipogenic, and chondrogenic commitment of BMPC were also studied. BMPC were obtained from animals of all experimental groups and incubated with lineage-specific differentiation culture media.

**Figure 6:**
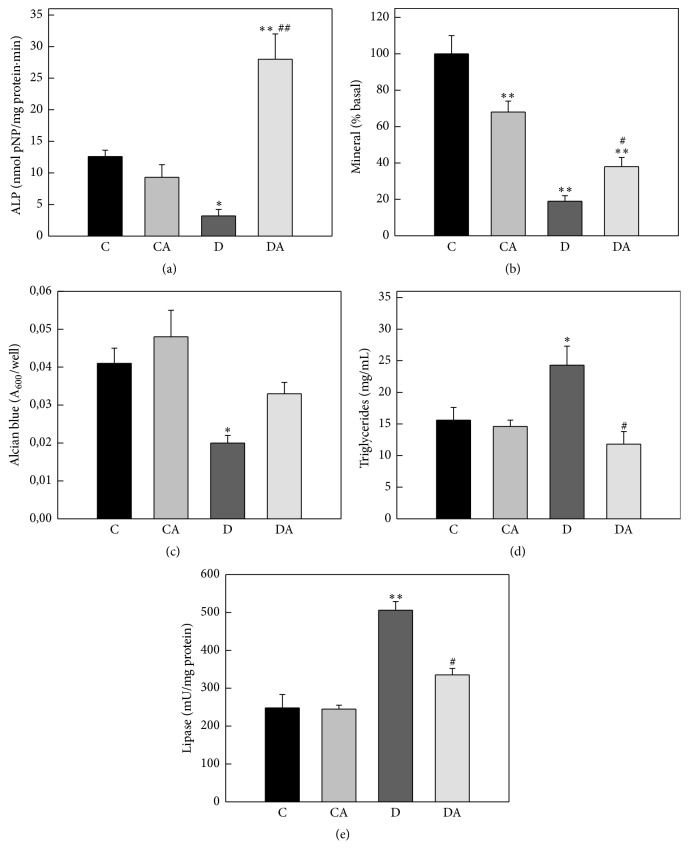
Effects of insulin-deficient Diabetes and oral treatment with alendronate on BMPC osteogenic, chondrogenic, and adipogenic potential. Diabetic and nondiabetic (control) animals were either left untreated (groups D and C) or treated for two weeks with 1 mg/kg/day of alendronate in their drinking water (groups DA and CA). BMPC were obtained from animals of all experimental groups and initially cultured in a basal medium. After confluence, BMPC were replated and submitted to lineage-specific culture conditions: (a) an osteogenic medium for 15 days to evaluate alkaline phosphatase enzymatic activity; (b) an osteogenic medium for 21 days to determine extracellular mineral nodule deposits; (c) a chondrogenic medium for 21 days to quantitate GAGs-associated Alcian blue staining; and an adipogenic medium for 10 days to measure intracellular triglyceride accumulation; (d) and lipase enzymatic activity (e). Results are expressed as the mean ± SEM. ^*∗*^ *p* < 0.05 versus C; ^*∗∗*^ *p* < 0.001 versus C; ^#^ *p* < 0.05 versus D; ^##^ *p* < 0.001 versus D.

As can be seen in Figure 6, Diabetes induced a significant decrease in markers of BMPC osteogenic and chondrogenic potential and an increase in their adipocytic induction. Specifically, BMPC obtained from diabetic animals (versus nondiabetic untreated controls) showed (a) lower ALP activity and extracellular mineral deposits (after 15 and 21 days of osteogenic differentiation, Figures 6(a) and 6(b)), (b) a decrease in glycosaminoglycan production (after 21 days of chondrogenic differentiation, Figure 6(c)), and (c) increases in both intracellular triglyceride deposits and lipase enzymatic activity (after 10 days of adipogenic differentiation, Figures 6(d) and 6(e)). Oral treatment of animals with alendronate partially or totally prevented all of these Diabetes-induced dysregulatory effects on BMPC phenotypic commitment (Figures 6(a)–6(e)).

## 4. Discussion

Diabetes mellitus (DM) and osteoporosis constitute an important burden for health care systems worldwide. Over the past 20 years a considerable body of experimental and clinical evidence has accumulated, pointing to an association between DM and bone abnormalities that include osteopenia, osteoporosis, and/or an increased incidence of low-stress fractures [29, 30]. This Diabetes-induced bone condition, also termed diabetic osteopathy, is believed to be partly due to accumulation of AGEs in the bone matrix. AGEs form inadequate and irreversible cross-links between collagen molecules, altering the biomechanical properties of bone. AGEs also depress the functionality of osteoblasts and osteoclasts via recognition by their receptor RAGE, potentially inducing a decrease in bone turnover that can further increase AGEs accumulation [15, 16]. A deleterious effect of AGEs on bone marrow cells has also been demonstrated: an excess of these end products (or of closely related advanced oxidation protein products) has been shown to increase the apoptosis and decrease the migration, proliferation, and osteogenic differentiation of BMPC while increasing their adipogenic potential [31–34]. In addition, we and other researchers have found that BMPC isolated from diabetic rats show an increase in their adipocytic potential and a decrease in their bone-forming capacity that correlate with alterations observed in long-bone histomorphometry and pQCT analysis [35, 36]. The* in vitro* results of our present study are compatible with those previous reports; we have found that AGEs (at doses similar to those found on proteins from skin and serum of diabetic patients) [37] decrease the osteogenic and chondrogenic differentiation while increasing the adipocytic commitment of BMPC obtained from control (nondiabetic untreated) rats. In addition, our present* in vivo* and* ex vivo* results confirm that the induction of insulin-deficient Diabetes in rats impairs long-bone microarchitecture, while disrupting BMPC multiphenotype potential (decrease in osteogenesis and chondrogenesis and increase in adipogenesis).

Alendronate is the most widely used agent for treatment of postmenopausal osteoporosis. This N-BP with high affinity for bone matrix shows mostly antiresorptive effects through its inhibitory action on the mevalonate pathway of osteoclasts. By this mechanism it reduces osteoclastic stress fiber and focal adhesion density, leading to a disruption of the actin cytoskeleton [38, 39]. However, low doses of alendronate have also been shown to stimulate osteoblast function* in vitro* [20, 40] and to modulate BMPC phenotypic induction by increasing osteogenesis and decreasing adipogenesis [41–44]. Our present results are in agreement with these previous studies:* in vitro* exposure of BMPC to alendronate alone (at doses that have been reported in the serum and resorption lacunae of patients treated with this N-BP) [45–47] increased their osteoblastic differentiation.

It is generally accepted that treatment with alendronate increases bone mineral density (BMD) and decreases fracture incidence; however, it also reduces bone tissue remodeling that could potentially lead to an accumulation of microdamage, adversely affecting energy dissipation capabilities and overall toughness of bone [48]. This reduction in bone remodeling would also be expected to increase accumulation of AGEs, and this was investigated by other researchers in nondiabetic dogs treated for a year with different doses of alendronate. They found an increase in bone brittleness and in bone matrix accumulation of AGEs at high doses of alendronate, but not at doses equivalent to those used for the treatment of postmenopausal osteoporosis [48]. Since Diabetes* per se* may also decrease bone turnover, the treatment option of diabetic osteopathy with alendronate must be closely analyzed in order to determine its pertinence and efficacy (particularly when long-term use of this N-BP is being considered). Through* in vitro* studies, we have previously demonstrated that while alendronate is able to prevent the antiosteogenic and proapoptotic effects of AGEs on osteoblasts [20], AGEs and alendronate potentiate each other to inhibit osteoclastic recruitment and activity [39]. In a clinical setting, two studies support the use of alendronate in diabetic patients: on one hand, a 3-year treatment with alendronate was associated with increased BMD in older women with type 2 Diabetes mellitus [17], whereas treatment with this N-BP for 5 years inhibited the decrease in radial BMD induced by Diabetes [18]. On the other hand, other researchers have warned that postmenopausal women with type 2 Diabetes could be resistant to long-term treatment with alendronate [19].

In view of this controversy, we have proposed the main hypothesis of the present study which is as follows: the deleterious changes in BMPC phenotypic commitment induced by AGEs accumulated in the bone matrix of an individual with Diabetes can be prevented by the presence of therapeutic doses of alendronate in the bone marrow microenvironment. In addition, these proosteogenic effects of alendronate on BMPC multilineage potential, in the context of diabetic osteopathy, will improve bone microarchitecture and functionality. Our present* in vitro* results support this hypothesis: we have found that exposure of control BMPC to AGEs induces a decrease in their expression of the osteoblast-specific transcription factor Runx2, type I collagen secretion, ALP activity, and extracellular mineral nodule deposition. However, when AGEs were coincubated with alendronate this antiosteogenic effect was completely abrogated. Similar results were observed when BMPC were induced to differentiate into chondrocytes: incubation with AGEs decreased their chondrocytic commitment (i.e., GAG production), whereas coincubation of AGEs with alendronate prevented this deleterious effect. On the contrary, the adipocytic differentiation of BMPC was potentiated by AGEs (increased expression of the proadipogenic regulator, PPAR-*γ*, of intracellular triglycerides and of lipase activity), an effect that was inhibited when AGEs were coincubated with alendronate.

This study's hypothesis is also supported by the results of our* in vivo* and* ex vivo* experiments. As stated above, development of partially insulin-deficient Diabetes in rats induced significant alterations in the microarchitecture of long bones (decreases in femoral metaphysis trabecular bone area, osteocyte density, and osteoclast TRAP activity and an increase in bone marrow adiposity). In addition, BMPC isolated from femora of diabetic rats showed a decrease in their osteogenic and chondrogenic potential and an increase in their capacity for adipogenesis. However, oral treatment of diabetic animals with alendronate prevented all of these Diabetes-induced effects on femoral microarchitecture and BMPC multilineage progression.

Intracellular ROS production is known to mediate various RAGE-dependent effects of AGEs. In an attempt to define molecular mechanisms that could be operating for both AGEs and BPs, in our present study we evaluated the intracellular production of ROS* in vitro,* with control BMPC, and found that both vitamin E and alendronate completely curbed the AGEs-induced increase in ROS production. These results are in agreement with our previous studies with osteoblasts [20] and with reports by other authors using human umbilical vein endothelial cells [49, 50]. RAGE-dependent intracellular ROS production is a well-known signal transduction mechanism that leads to NF-KB activation and expression of various subsets of genes in different cell types. However, an excessive increase in ROS production due to AGEs accumulation could induce an intracellular prooxidative state, promoting an increase in advanced oxidation protein products and ultimately cell apoptosis. This may constitute an indirect deleterious effect of AGEs on BMPC, contributing to the decrease in bone formation that has been described in experimental models of Diabetes. The* in vitro* results for ROS, in our present study, provide a possible mechanism of action for the* in vivo* preventive actions of alendronate on the antiosteogenic effects of experimental Diabetes: AGEs accumulated on bone matrix proteins exposed to hyperglycaemia can induce ROS in nearby BMPC, an effect that could be prevented by therapeutic doses of alendronate in the bone marrow microenvironment.

In conclusion, our results suggest that extracellular AGEs accumulation can alter BMPC fate by an increase in intracellular ROS production, which in turn modulates the expression of lineage-specific regulatory factors such as Runx2 and PPAR*γ*, decreasing the osteoblastogenic and chondrogenic potential of BMPC in favour of adipogenesis. Importantly, all these AGEs-induced effects on BMPC can be prevented* in vitro* by cotreatment with therapeutic doses of alendronate, effects that are also operative* in vivo*. All in all, our present findings support the results of previous reports that show a beneficial action of alendronate in patients with Diabetes mellitus and osteoporosis.

## Figures and Tables

**Table 1:** Biochemical parameters in nonfasting serum obtained from animals of all experimental groups.

Serum parameter	Control	Control + alendronate	Diabetic	Diabetic + alendronate
Glucose [mg/dL]	168 ± 6	175 ± 9	453 ± 40^*∗*^	380 ± 33^#&^
Triglycerides [mg/dL]	62 ± 7	54 ± 4	134 ± 42^#^	128 ± 19^#^
Fructosamine [*μ*mol/L]	120 ± 15	135 ± 13	190 ± 12^%^	210 ± 16^%^
Cholesterol [mg/dL]	37 ± 0.8	37 ± 1	51 ± 5^#^	56 ± 7^#^

Results are expressed as the mean ± SEM, *n* = 8.

Differences versus C: ^#^ *p* < 0.05; ^%^ *p* < 0.01; and ^*∗*^ *p* < 0.001.

Differences versus D: ^&^ *p* < 0.01.
